# Staged Percutaneous Management of Pulmonary Atresia and Intact Interventricular Septum: Stretching the Limits

**DOI:** 10.1155/2023/9709227

**Published:** 2023-02-01

**Authors:** Sonia A. El-Saiedi, Wael A. Attia, Baher M. Hanna, Mahmoud O. Aboudeif, Rania Zakaria, Mohamad Abd ElMeguid, Ashraf Abd El Reheem, Reda Abuelatta

**Affiliations:** ^1^Pediatric Department, Division of Pediatric Cardiology, Cairo University, Giza, Egypt; ^2^Radiodiagnostics Department, Cairo University, Giza, Egypt; ^3^Cardiology Department, Cairo University, Giza, Egypt; ^4^Anaesthesia Department, Cairo University, Giza, Egypt; ^5^Senior Cardiology Consultant, Cardiology Centre, Medina, Saudi Arabia

## Abstract

**Aims:**

Pulmonary atresia with intact ventricular septum (PA/IVS) can be treated by catheter-based interventions and complemented by various surgical procedures. We aim to determine a long-term treatment strategy to enable patients to be surgery free, depending solely on percutaneous interventions.

**Methods and Results:**

We selected five patients from among a cohort of patients with PA/IVS treated at birth with radiofrequency perforation and dilatation of the pulmonary valve. Patients had reached a pulmonary valve annulus of 20 mm or larger on their biannual echocardiographic follow-up, with right ventricular dilatation. The findings, together with the right ventricular outflow tract and pulmonary arterial tree, were confirmed by multislice computerised tomography. Based on the angiographic size of the pulmonary valve annulus, all patients were successfully implanted with either Melody® or Edwards® pulmonary valves percutaneously, regardless of their small weights and ages. No complications were encountered.

**Conclusion:**

We managed to stretch the age and weight limitations for performing percutaneous pulmonary valve implantation (PPVI): interventions were attempted whenever a pulmonary annulus size of >20 mm was reached, which was rationalised by the prevention of progressive right ventricular outflow tract dilatation and accommodating valves between 24 and 26 mm, which is enough to sustain a normal pulmonary flow in adulthood.

## 1. Introduction

Pulmonary atresia with intact ventricular septum (PA/IVS) includes a group of heterogenous anomalies characterized by complete membranous or muscular atresia of the pulmonary valve (PV) and an intact interventricular septum. A patent foramen oval or an atrial septal defect is always present, together with an abnormal tricuspid valve and right ventricle (RV), with variable degrees of hypoplasia. A normal left-sided heart and aorta are present in 98% of cases [1, 2].

Initial intervention by aggressive decompression of the RV in the neonatal period can be achieved in most cases, except for those with RV-dependent coronary circulation [3]. In cases with a tricuspid valve annulus *Z*-score larger than −3, we have adopted the use of an initial intervention consisting of PV perforation and dilatation, whereas for those with bipartite RV and a *Z*-score from −2 to −3, ductal stenting is added [4].

There is a paucity of information in the literature on the fate of the PV after perforation. The few published data have described RV growth [5, 6] and the need for reintervention, either surgical or percutaneous [7, 8]. Nonetheless, no attempts have been made to describe the optimal timing for PV replacement while taking into consideration the young age of these children.

In recent years, percutaneous pulmonic valve implantation (PPVI) has been developed as an attractive alternative to open surgical valve replacement, which also expands the scope from just dysfunctional valves and conduits to treatment of wide native right ventricular outflow tracts (RVOTs) [9]. PPVI is well established in patients who weigh more than 30 kg, whereas very few reports have included patients weighing less than 30 kg with a minority of less than 20 kg, [10] using either the Melody® [11] valve or less commonly the Edwards SAPIEN valve [12, 13], in heterogenous groups of patients with RVOT dysfunction including conduits [14]. In patients with PA/IVS who have previously undergone radiofrequency perforation and balloon dilatation, we attempt early intervention via PPVI using the Edwards SAPIEN® or Medtronic Melody® valves.

## 2. Methods

Since September 2009, we have performed 101 cases of radiofrequency (RF) perforation and dilatation of the PV to manage cases with PA/IVS with Z-scores larger than −3, with a success rate of ∼60% [15]. Since then, successful cases have been followed up every 6 months to monitor RV growth and the development of the PA and branches, in addition to the degree of pulmonary regurgitation and/or stenosis.

Ten cases were selected from our cohort with ages between 7 and 10 years, who were treated initially in the neonatal period, whose pulmonary annulus by echocardiography reached 20 mm or greater. Multislice computerised tomography (MSCT) was performed in nine cases. The parents of one patient refused to provide consent to the MSCT and the PPVI. Two cases were determined to have pulmonary annulus of 17 and 18 mm by MSCT; accordingly, we decided to continue following these cases every 6 months. Another two cases were found to have some neurologic deficit and were excluded from PPVI for the time being. Five cases were found to be eligible for PPVI.

## 3. Procedure

A written informed consent was obtained from each parent/legal guardian for the intervention and use of data.

All cases were conducted via the femoral vein. The standard technique [16] was adopted in all cases, whether implanting the Melody or the Edwards SAPIEN valve. Balloon interrogation and coronary artery compressibility were conducted using a 1 : 1 balloon size with contrast injection through the side arm of the 16F kink-resistant long sheath (Cook Medical, Bloomington, IN, USA) to the RVOT to ensure adequate sealing with the selected balloon size.

For all Melody cases, RVOT stenting is the standard of care, to abort the distal valve stent fracture. Although the Edwards valve can also be considered, it is safe to implant the Edwards valve in RVOT dysfunction without a landing stent [17].

Procedures were performed under general anaesthesia with endotracheal intubation and muscle relaxant along with continuous arterial blood pressure monitoring and ACT. A backup surgical team was ready for any emergency.

After the procedure, patients remained in the intensive care unit for 1 day and were then discharged on dual antiplatelet therapy (aspirin and clopidogrel), which was continued for at least 6 months.

## 4. Results

All patient's data are summarized in Table 1 including age, weight, and stents used plus the chosen valve.

**Figure 1:**
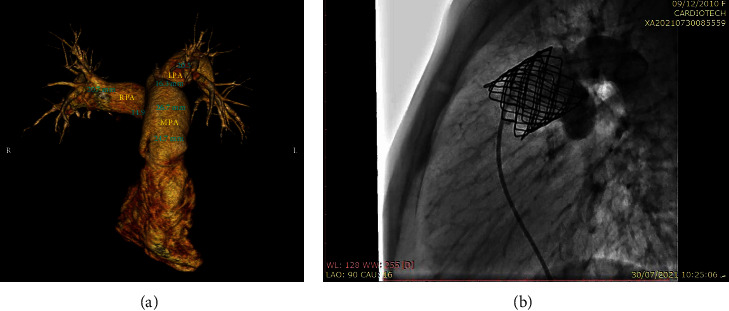
Case 1. (a) MSCT showing the tubular-shaped MPA, PA annular size. and distance to bifurcation; (b) CP Stent 8Z 34 mm with melody 22 mm.

In case 1, MSCT revealed a tubular main PA (MPA) with a PA annulus of about 24 mm (Figure 1(a)). This patient was prestented with a 39 mm CP Stent, and the valve was Melody size 22 mm and overinflated to 24 mm. The final injection showed a competent PV (Figure 1(b)).

**Figure 2:**
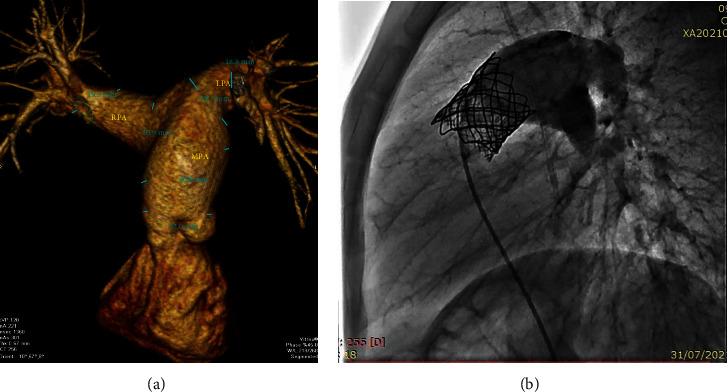
Case 2. (a) MSCT showing a dilated MPA compared with annulus. (b) CP Stent 39 mm with the Edwards 23 mm valve well placed.

Case 2 had an aneurysmal MPA on MSCT (Figure 2(a)) with a short MPA to bifurcation. For this reason, we chose to implant a 23 mm Edwards valve without prestenting. The final injection showed an excellent result (Figure 2(b))

**Figure 3:**
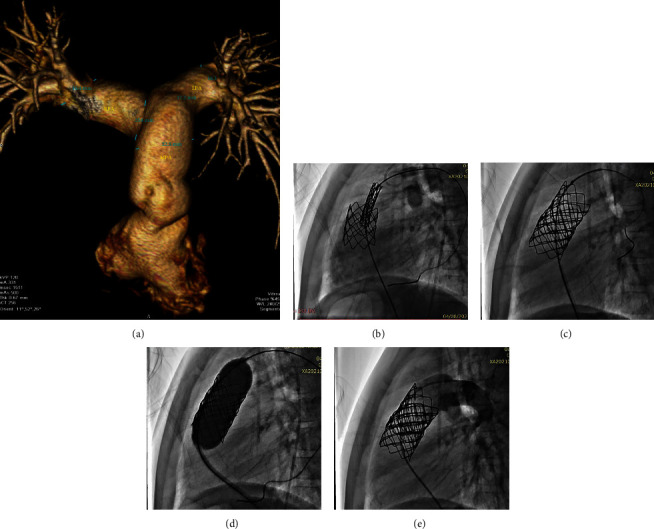
Case 3 (nightmare case). (a) MSCT showing a tubular-shaped MPA and distance to bifurcation. (b) Stent and Melody valves were displaced downward in RVOT. (c) Attempting to push the valve and stent back upward using a balloon. (d) Placing a second stent within the stent/valve system and sacrificing the first valve. (e) End result with the second Melody 22 mm PV placed higher in the MPA and well functioning.

Case 3 MSCT showing a tubular-shaped MPA (Figure 3(a)). On delivering the valve, we noted that the stent was displaced downwards. Aiming at fixing this malposition, we deployed the valve in the upper third of the stent, but the whole system was displaced further downwards (Figure 3(b)). We attempted to push it upward using the inflated balloon (Figure 3(c)), and although the MPA angiogram showed a competent valve, the position was not secure. We decided to sacrifice the valve by placing another longer 39 mm CP Stent higher in the MPA (Figure 3(d)) followed by another valve within. The final angiogram showed a competent valve within a stable, secure position (Figure 3(e)).

**Figure 4:**
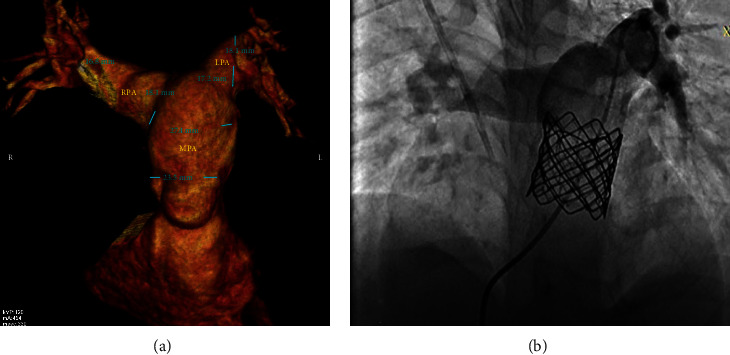
Case 4. (a) MSCT showing aneurysmal MPA. (b) Final injection after installing the Edwards SAPIEN valve.

**Figure 5:**
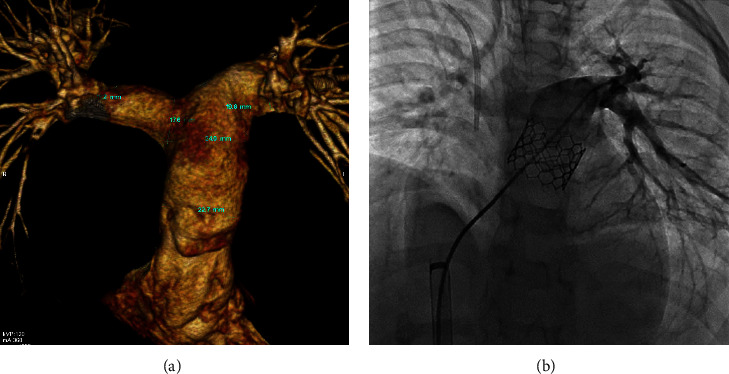
Case 5. (a) MSCT showing dilated MPA. (b) Final injection after installing the Edwards SAPIEN valve.

Both cases 4 and 5 were presented with a 39 mm CP Stent. As shown in MSCT, both cases had a large MPA of about 25 mm and required a 26 mm Edwards SAPIEN valve. They showed an excellent position and a competent valve upon completion (Figures 4 and 5).

## 5. Discussion

Restoration of biventricular physiology with good function is the ultimate goal of treatment strategies for congenital heart defects, whenever possible. With advances made in science, technology, and experience, an increasing number of patients who were doomed for univentricular repair are being relocated to the biventricular group with good results. In our 10-year experience with neonates who have pulmonary atresia and intact interventricular septum, [15] we have established radiofrequency perforation and dilatation of the atretic PV as the gold standard for treatment instead of PDA stenting and the univentricular pathway and/or surgery. Because of pulmonary regurgitation, with or without variable degrees of residual annular stenosis, the RVOT and MPA in addition to RV become dilated in the long term, analogous to what is classically observed following the repair of tetralogy of Fallot with a transannular patch; PPVI is gaining more popularity in these patients with results superior to that of surgical replacement [18, 19].

The indications for PPVI have expanded from RVOT conduits or patches to include native dysfunctional outflow tracts [20]. Indeed, we need to overstep the boundaries with the resolution of improving patients' quality of life and minimising interventions [21].

Under this directive, we have attempted to implant competent PVs in our paediatric patients who have PAs that have reached sizes suitable for the PPV without waiting to reach the age/weight guidelines. PPVI has been performed previously for paediatric patients with repaired RVOTs [11, 12, 22].

Very few reports have included cases as young as 3.8 years and 13.5 kg using the Melody valve, and these are primarily reported in cases with repaired Fallot or conduits [10, 11]. Few cases with PA/IVS have been included in previous reports [12, 13, 22] to be implanted with the Melody valve or less frequently with the SAPIEN valve [12, 17]. We have limited data on those cases whether treated initially surgically or percutaneously; the reports also did not describe the following factors in this particular cohort: the age, weight, or size of the valve used. Shahanavaz et al. in their big series, including 33 cases with PA/IVS, have concluded that the procedure was less likely to be successful in patients less than 30 kg [13].

Waiting for such children to reach the minimal weight of 30 kg exposes them to develop aneurysmally dilated PAs that exceed the 30 mm limit for any of the currently available PPVs and also puts the RV at risk of progressive dilatation and dysfunction, which can directly affect the outcome, as we have learned from the long-term outcomes of repaired tetralogy of Fallot [23, 24]. These results have led to a recommendation of cut-off values of 139 mL/m^2^ and 75 mL/m^2^ for indexed RV end-diastolic volume and RV end-systolic volume, respectively [25].

**Figure 6:**
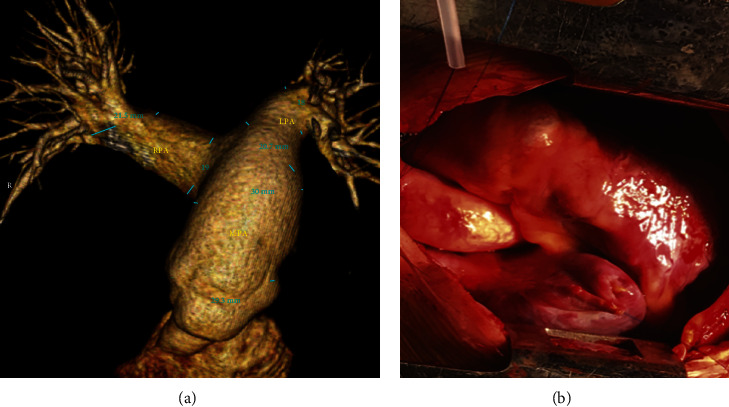
Surgical case (a) MSCT showing aneurysmally dilated MPA and RVOT with PA annulus >30 mm. (b) Intraoperative view with the PA size twice that of the aorta.

Optimising the age for PV intervention has always been a difficult task. On the one hand, earlier repair is associated with better long-term outcomes [26, 27] and avoids undue excessive enlargement of the RVOT/PA that makes interventions more complicated, and on the other hand, delayed interventions maximise the chances of feasibility of transcatheter intervention and delay possible reinterventions. However, we have noticed in our cohort of patients with PA/IVS that the RVOT enlarges quickly beyond expectations. In fact, at the time we focused on weight rather than PA size, one case from our series reached 31 kg at 9 years of age and had to undergo surgical repair, with a hugely dilated PA twice the aorta, requiring surgical refashioning of the PA and RVOT with implantation of a St Jude Epic 25 mm valve in addition to tricuspid valve repair (Figure 6). Thus, weighing risk/cost versus benefits, we have opted to intervene at any age based on the PA size (i.e., whenever the adult size of >20–22 mm is reached).

We did not depend on the RV volume or index by magnetic resonance but rather the MSCT angiography data, including the PA annular diameter, length from the annulus to bifurcation, and the MPA morphology (tube-like or aneurysmal). By this, we have challenged the well-established indications of the American College of Cardiology/American Heart Association European and Canadian guidelines, which are symptoms of RV failure requiring pharmacologic therapy, severe RV hypertension with an RV/LV pressure >0.7, peak and mean Doppler gradients across the PV of >50 mmHg and 30 mmHg, respectively, indexed RV end-diastolic volume >160/mL/m^2^ or RV end-systolic volume >80 mL/m^2^, RV end-diastolic volume ≥ two times the LV end-diastolic volume, reduced RV ejection fraction <0.40–0.45, and a QRS duration of ≥180 ms [28–30].

Age also limits the procedure in the required larger veins to accommodate large sheaths up to 20F. Over time, companies have provided valves up to 30 mm that can go in 16F sheaths, which have helped the reduction in age limits. In fact, as a result of neonatal intervention, we have encountered stenosis of the right iliac vein in two cases that would not allow the long sheath, and we had to shift to the left side. We have been able to reduce the age and weight limits to as young as 7 years and 20 kg. Martin et al. have favoured the use of the jugular vein with the Melody valve implantation in weights less than 17 kg [11]; others have utilized a hybrid approach for the younger patients [13].

We acknowledge that some operators have attempted to overcome the large RVOT by bilateral branch PA valve implantation [31]. As inspiring as this seems, we think that it is better to obtain a more physiological solution to prevent RVOT from further dilatation and the potential-related complications including arrhythmias, as well as to prevent the need to reintervene in the future on two degenerated valves rather than one in a more complex setting, as the RVOT anatomy might become more distorted.

Dysfunctional RVOT is frequently tricky to treat, especially with bulky sheaths and delivery systems. To be able to drive the system forward without loss of the wire position, manipulations need to be very shrewd. We encountered two instances in which we had to railroad the RVOT using a Backup Meier Guidewire (Boston Scientific, Marlborough, MA, USA) to properly interrogate the RVOT and to aid passage of the delivery system.

When attempting to replace the valves in the pulmonary position, prestenting is typically performed to provide a secure landing zone in the dysfunctional RVOT caused by a degenerated homograft or a transannular patch and also to protect the valve frame from fracture by the calcified RVOT or the fractured surgical valve stent [32]. A rationale for not performing prestenting is having a native annulus that could securely hold the valved stent: in one of our cases, we were able to implant an Edwards valve without prestenting, similar to the report by Morgan et al. [17]. For this approach, the PA should not be excessively dilated; also, it could be of merit in cases with a short annulus to bifurcation ratio.

Currently available valves include the Medtronic Melody (Medtronic, Minneapolis, MN, USA) and SAPIEN (Edwards Lifesciences, Irvine, CA, USA), although the options soon will become more diverse.

Melody valves, which are approved by the US Food and Drug Administration for the pulmonary position, consist of a manually sewn bovine jugular vein to a platinum-iridium bare metal stent. These valves are easier to manipulate through the RVOT but have the disadvantage of the limited sizes of 16–18 mm that can be expanded up to 22 mm [33]. The Medtronic HARMONY VALVE soon will become popular, as it can reach 30 mm with smaller sheath sizes.

SAPIEN valves are bovine pericardial tissue valves that are hand sutured to stainless-steel radiopaque stents, available in sizes 23, 26, and 29 mm. They are used after CE certification in the pulmonary position and have been previously reported in adult patients [34–38]. The delivery system is more complex and designed for aortic valve implantation, resulting in significant difficulty when negotiating the RVOT because of the curves involved, especially in smaller hearts. As an alternative to overinflating the Melody valve and exposing it to the risk of regurgitation, we prefer the use of this valve in larger RVOTs. The next-generation Edwards SAPIEN XT valves have a cobalt chromium frame, and SAPIEN3 is designed to minimise perivalvular leak, as it is equipped with an outer pericardial skirt [39].

The PULSTA (TaeWoong Medical Co, Gyeonggi-do, South Korea) self-expandable valve ranging in size from 18 to 32 mm has the advantage of having flared ends that can adapt to large RVOTs and different geometries and also includes a low-profile introducer system [40, 41].

To conclude, we report our experience with five paediatric cases of PA/IVS weighing less than 25 kg who were fully treated percutaneously: initially as neonates by radiofrequency perforation and PV dilatation and completed by PPVI using the Edwards and Melody valves. We advocate for early intervention whenever the PA reaches the suitable size, regardless of age and/or weight.

## Figures and Tables

**Table 1:** Case data.

Cases	Radiofrequency age (days)	Age at PPVI (years)	Weight at PPVI (kg)	PA annulus, distal size	Length to bifurcation	MPA shape	Prestent type	Prestent length	Valve size	Valve used
1	21	10	24	2.5–2.4	3.5	Tubular	CP 8Z	34	22	Melody
2	29	8.5	24	2.2–3.0	3.6	Aneurysmal	—		23	Edwards SAPIEN
3	17	7.5	25	2.3–2.2	2.2	Tubular	CP8Z	34 and 39	22 and 22	2x melody
4	40	8	30	2.2–2.4	2.4	Tubular	CP8Z	39	26	Edwards SAPIEN
5	12	8	22	22.5–2.7	3.0	Conical	CP8z	39	26	Edwards SAPIEN

## Data Availability

The data that support the findings of this study are available from the corresponding author upon request.
